# Semi-Automatic Lab-on-PCB System for Agarose Gel Preparation and Electrophoresis for Biomedical Applications

**DOI:** 10.3390/mi12091071

**Published:** 2021-09-02

**Authors:** Jesús David Urbano-Gámez, Francisco Perdigones, José Manuel Quero

**Affiliations:** Electronic Engineering Department, University of Seville, 41004 Sevilla, Spain; jurbano1@us.es (J.D.U.-G.); quero@us.es (J.M.Q.)

**Keywords:** lab-on-PCB, electrophoresis, biomedical applications, agarose

## Abstract

In this paper, a prototype of a semi-automatic lab-on-PCB for agarose gel preparation and electrophoresis is developed. The dimensions of the device are 38 × 34 mm${}^{2}$ and it includes a conductivity sensor for detecting the TAE buffer (Tris-acetate-EDTA buffer), a microheater for increasing the solubility of the agarose, a negative temperature coefficient (NTC) thermistor for controlling the temperature, a light dependent resistor (LDR) sensor for measuring the transparency of the mixture, and two electrodes for performing the electrophoresis. The agarose preparation functions are governed by a microcontroller. The device requires a PMMA structure to define the wells of the agarose gel, and to release the electrodes from the agarose. The maximum voltage and current that the system requires are 40 V to perform the electrophoresis, and 1 A for activating the microheater. The chosen temperature for mixing is 80 ${}^{\circ }$C, with a mixing time of 10 min. In addition, the curing time is about 30 min. This device is intended to be integrated as a part of a larger lab-on-PCB system for DNA amplification and detection. However, it can be used to migrate DNA amplified in conventional thermocyclers. Moreover, the device can be modified for preparing larger agarose gels and performing electrophoresis.

## 1. Introduction

Nowadays, the development of microfluidic devices using printed circuit board (PCB) substrates has been the subject of increasing research [1,2]. These devices are named lab-on-PCBs (LoP), and they can be considered to be a part of lab on a chip devices (LoCs). The use of PCB substrates has interesting advantages for biomedical applications [3], such as the possibility of easy integration of electronics and microfluidics with sensors and actuators in a single platform; commercial availability and low cost production, to name a few. It is important to emphasise that lab-on-PCBs can include several laboratory tasks, such as micromixing, sensing, chemical reactions and heating in a device with the dimensions of a credit card.

Apart from the PCB substrate, lab-on-PCBs was fabricated using different rapid prototyping materials, such as SU-8 [4] and PDMS [5,6]. However, the use of thermoplastics for the microfluidic component of the device is a more interesting option from the point of view of the market. In this respect, the device fabrication can be intended as mass production, that is, thermoplastic fabrication using hot embossing or injection molding, and the PCB can be ordered to specialised companies. These characteristics make lab-on-PCBs an attractive choice, due to their high potential for commercialisation [1,2].

Up to now, lab-on-PCBs include several biomedical applications, for example, for detecting cell viability [7], molecular diagnosis [8], organotypic cultures [9], or electrolytes detection [10]. Focusing on electrophoresis-based applications, capillary electrophoresis (CE) is the most usual technique in lab on chip [11]. This is because this kind of electrophoresis may be automated, and direct quantification is possible. Among others, CE is used for the separation of monosaccharides, oligosaccharides, and polysaccharides [12], separation of proteins [13] and for applications in life sciences in general [14]. On the other hand, conventional electrophoresis continues being a robust and very used method [15] both for lab on a chip applications [16,17] and conventional laboratories [18].

Whatever the method, the electrophoretic gel (agarose or polyacrylamide) is manually fabricated in many laboratories. These methods require heating, time to achieve transparency and a curing time to solidify the material. The current methods suggested for many agarose manufacturers can be seen in [19,20,21], to name a few. As previously commented, the procedure is manual. In this respect, Erlenmeyer flasks or beakers are required. When the agarose is placed in the buffer, it is insoluble at room temperature. However, when the agarose solution is heated, the agarose particles increase the solubility and they become hydrated, and therefore go into solution. This heating is performed using a microwave at high power. Moreover, the suggested method to stop the mixing process is boiling and transparency. However, the transparency is subjective because it depends on the expertise of a technician, without an exact measure of the transparency. Furthermore, the process implies big equipment when compared to the functionalities that a lab on a chip can offer. On the other hand, there are companies, for instance Invitrogen^TM^ (Thermofisher Scientific), that supply the ready-to-use gel cartridge. In this case, the dimensions are fixed, they are not integrable on a lab on chip, nor are they customizable, and they are intended to be used in its reader. Moreover, the quantity of agarose for laboratories is limited to the commercial electrophoretic tanks dimensions. In this respect, new techniques are used for preparing the agarose gel [22]. The agarose gel electrophoresis is typically used to resolve RNA and DNA; polyacrylamide gel electrophoresis is used to separate proteins. Therefore, there is a wide variety of applications that are processed in laboratories.

The trend of the lab on chip developments includes the integration of these applications in a substrate with the dimensions of a credit card. In order to do so for qualitative PCR, the thermocycling, the agarose gel preparation, the electrophoresis and the detection have to be integrated in the same platform, with a process as automatic as possible. Regarding DNA amplification, many approaches have been reported [23,24,25]. Lab-on-PCB devices have also been developed, for example, for a three-temperature protocol [17] and for two temperatures [16]. All of them require the agarose gel preparation, using the typical procedure. Moreover, none of them have the preparation of the agarose integrated in the same platform. Regarding the electrophoresis on chip, there are many devices apart from CE [26,27], especially for polyacryamide gel. In these cases, photopatterning of polyacrylamide gels in glass or PDMS microfluidic devices is performed, in order to prepare the gel in the microchannels [28,29,30,31,32,33]. These devices demonstrate interesting biological applications, using materials fabricated with no low-cost processes. The device reported on [34] is used for analysing single-cell genomic damage. It is an interesting device fabricated using soft lithography for agarose gel and a SU-8 mold. The fabrication process of this device is manual, using rapid prototyping materials. In this case, the integration of the conventional electrophoretic method for DNA migration on lab on chip is challenging due to the low automation of the method. Finally, the detection method in an important component of the whole system. In this respect, inexpensive and single-use lab on a chip devices are not intended to include the detection system. These systems are composed of expensive components, especially for absorbance or fluorescence, for example, photodiodes, photomultipliers or phototransistors. These single-use lab on chips require a reader to perform the detection.

In this paper, a semi-automatic and disposable lab-on-PCB for preparing agarose gel and for performing electrophoresis is described. It includes a microheater to achieve the mixing by increasing the solubility of the agarose in the TAE buffer (Tris-acetate-EDTA buffer). Regarding the buffer, TBE buffer (Tris-borate-EDTA buffer) could be used as well. The device has integrated sensors, such as a thermistor to control the temperature and light-dependent resistor to ensure the required transparency. In addition, it includes an interdigitated capacitive sensor to detect the filling of the cavity with the liquid. All these sensors and actuators are connected to a microcontroller, which governs the working of the lab-on-PCB. The device testing includes preparing the agarose gel using the device, and performing the electrophoresis. The device implies low-cost and single-use characteristics, fast analysis, low sample consumption and integration capability. It is intended as an integrable functional module of a more complex system for DNA amplification and detection, or even a device itself to perform more controllable electrophoresis in conventional laboratories, minimizing the human factor.

## 2. Lab-on-PCB Brief Description

In this section, the function of the lab-on-PCB is briefly described. Then, in Section 3, the sensors and actuators are described for a complete understanding of the device.

**Figure 1:**
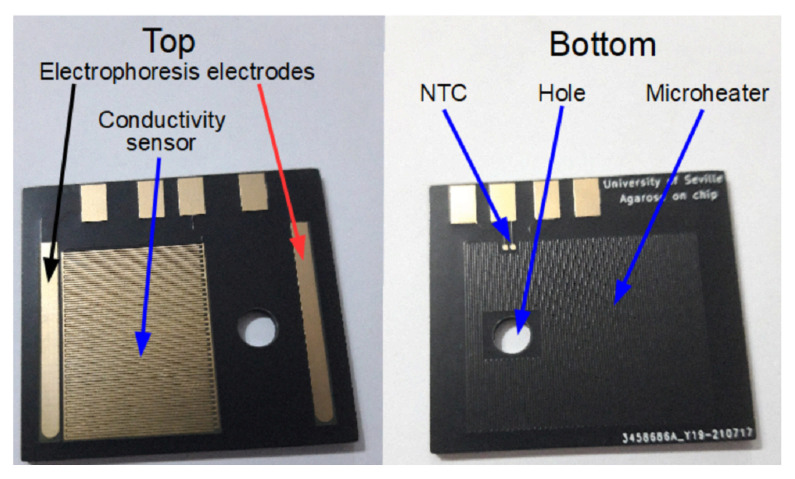
Printed circuit board substrate for agarose mixing and curing, and electrophoresis. (**Left**) Conductivity sensor and electrophoresis electrodes are shown. (**Right**) The microheater and the thermistor can be seen. The dimensions of the device are 38 × 34 mm${}^{2}$.

The PCB substrate has two functional components; Figure 1. The first one is intended to control the agarose mixing and curing, and the second one to perform the electrophoresis. The dimensions of the PCB are 38 × 34 mm${}^{2}$.

Regarding the preparation of the agarose, the device includes a microheater in order to mix the agarose with the TAE buffer. Typically, the mixing is considered finished when the agarose is transparent enough. Therefore, the degree of transparency needs to be monitored. In order to do so, a light dependent resistor (LDR) sensor is used. In addition, the mixing temperature is measured with an integrated surface mounted device (SMD) thermistor to perform the control of temperature. On the other hand, the device includes a conductivity sensor in order to detect the filling of the cavity with the liquid to start the automatic process. These steps, that is, the mixing and the curing of the agarose are governed by a microcontroller. Finally, the electrophoresis is performed, using two gold electrodes integrated in the same PCB substrate.

**Figure 2:**
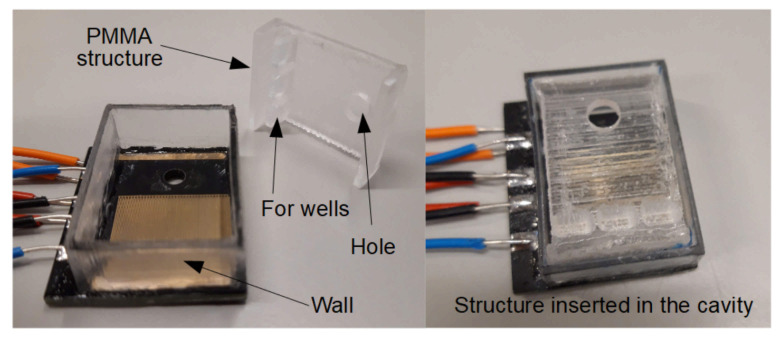
The lab-on-PCB with the thermoplastic wall and the auxiliary structure are shown.

The device has polymethylmethacrylate (PMMA) walls, which limit the area of the fabricated agarose gel. This part has an auxiliary structure to define both the wells in the agarose gel and the volume above the electrodes; Figure 2. The auxiliary structure has to be inserted in the cavity before the filling with the TAE buffer.

**Figure 3:**
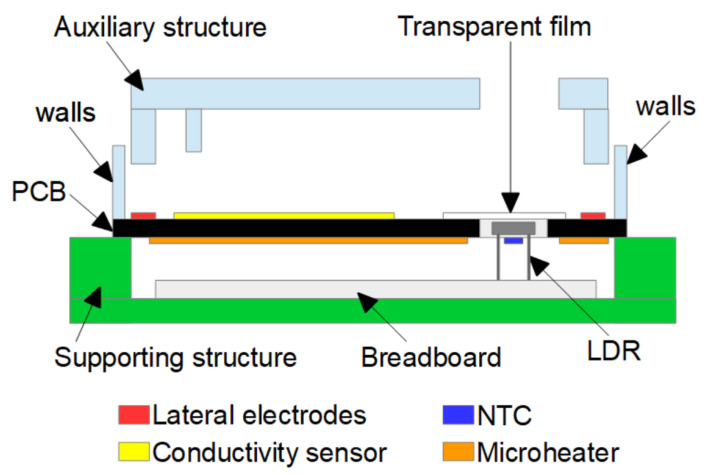
The cross-sectional view of the prototype is shown. The supporting structure, the breadboard and the location of the sensors and the transparent film can be seen. The negative temperature coefficient (NTC) sensor is not under the light dependent resistor (LDR), it is in a different plane.

In order to clarify the assembly, a drawing of a cross-sectional view of the lab-on-PCB is shown in Figure 3. As can be seen, the transparent film is placed on the top side of the PCB substrate, and the LDR sensor is located below the transparent film. The detection system is not included because it is an independent part of the system.

**Figure 4:**
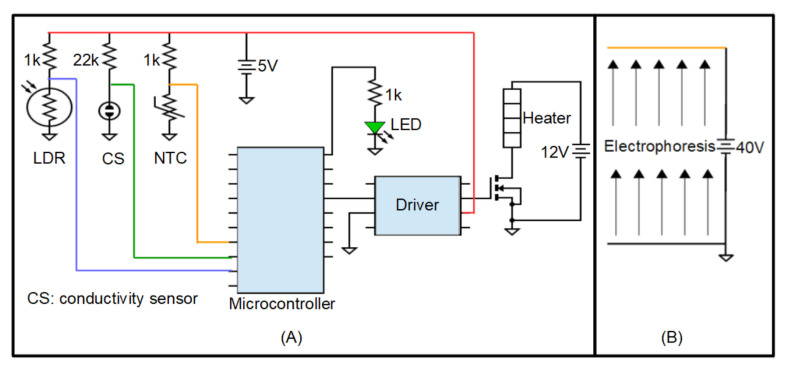
(**A**) Basic signal conditioning electronic circuit connected to the microcontroller. (**B**) The electrophoresis schematic circuit. The arrows indicate the direction of the migration.

The device requires a basic signal conditioning circuit in order to manage both the current along the microheater and the measure of the sensors outputs. The whole behaviour of the lab-on-PCB for agarose gel preparation is governed by a microcontroller. The schematic of the electronic circuit can be seen in Figure 4.

## 3. Sensors and Actuators Description

### 3.1. Thermal Actuation

The microheater is directly integrated in the bottom side of the PCB substrate during fabrication, and covered with the solder mask. It is performed by the commercial PCB manufacturer with a very good finish. This microheater is a copper serpentine with a length of 2.1 m, a width of 150 μm, a spacing between lines of 150 μm, and a thickness of 35 μm. The experimental resistance of the copper microheater is 7 Ω. The control of the temperature is performed using a SMD thermistor (part number: NXFT15XH103. Resistance = 10 kΩ, B-constant = 3380 K and package 0402, Murata Manufacturing Co., Ltd., Kyoto, Japan) which is placed close to the microheater. The temperature needs to be characterised previously because the temperature of the liquid over the microheater and the thermistor temperature is not necessarily the same. This characterisation is described in Section 5.

### 3.2. Optical Sensing

The PCB substrate has a hole with a diameter of 4.5 mm, covered with a transparent and PCR-compatible film (ThermalSeal^®^films, classic). The optical sensor is a LDR (NSL-19M51) with a diameter of 4.3 mm and a minimum light resistance of 20 kΩ. It is placed just below the transparent film of the PCB hole. As can be seen in Figure 2, the auxiliary structure has a hole to avoid the lack of transparency due to the CNC laser fabrication process. This hole is just above the PCB hole in order to create an optical path, which allows to measure the transparency. The values of the optical sensor have to be characterised to define the transparency at the end of both the mixing and the curing. This characterisation is described in Section 5.

### 3.3. Conductivity Sensing

Similarly to the microheater, the conductivity sensor is fabricated by the manufacturer company, in this case, in the top side of the substrate and covered with gold. This sensor is an integrated interdigitated transducer (IDT) with 74 electrodes with a width of 150 μm, a gap of 150 μm, and a length of 1.8 cm. The dimensions of the electrodes and the microheater are limited by the selected technology, but they can be reduced by increasing the fabrication cost. This sensor requires characterisation in order to define the value for detecting the TAE buffer. This characterisation is described in Section 5 together with the optical and thermal experiments.

### 3.4. Electrophoretic Actuation

The migration of the DNA is performed by actuation on the lateral electrodes. These electrodes are integrated during the fabrication process. They are copper electrodes covered with gold for reducing the oxidation during the electrophoresis process. The electrodes have a length of 2.8 cm, a width of 2 mm and a thickness of 35 μm.

## 4. Process Sequence

The steps of the process can be seen in Table 1 and are commented thereafter.

The device with the PMMA structure assembled can include the agarose powder (CSL-AG500 Cleaver Scientific, Rugby, Warwickshire, UK) over the surface before starting the process, in this case 100 mg for a 4 ml agarose gel (final concentration 2.5% *w*/*v*). The first step consists of filling the cavity with the agarose-TAE mixture with SYBRSafe DNA staining solution (S33102 ThermoFisher Scientific, Waltham, Massachusetts, USA) to perform the mixing, where the TAE buffer is (15558042 ThermoFisher Scientific). This filling is performed using a syringe pump (NewEra Pump Systems NE-1000), with a volume of 4 mL. The percentage of agarose can be modified by changing the TAE buffer volume, the quantity of agarose or both of them. This step is detected by the conductivity sensor, which sends the signal for starting the automatic process. The next step consists of disabling the conductivity sensor, supplying the required current to the microheater, and sensing the degree of transparency. Once the transparency is achieved, the third step takes place automatically, that is, the microheater is disabled in order to cool down the mixture. In this step, the LDR sensor continues to be enabled because the degree of transparency of the cured agarose is lower than the freshly mixed agarose. The automatic process finishes when the agarose is cured, after which the microcontroller activates a LED in order to inform that the process is finished, and disable the sensors. Finally, the following step is not automatic; it consists of removing the PMMA structure to define both the wells and the cavities to pour the TAE buffer. After this process, the device is ready to be loaded with the liquid to be migrated, using electrophoresis. The experimental results show the performance of the fabricated agarose gel for electrophoresis.

Once the agarose gel is cured and the PMMA structure is removed from the substrate, the next step consists of loading the wells with DNA. After that, the electrophoresis is performed, using an independent power supply of 40 V (the rest of the power supplies are switched off), for which the lateral gold electrodes are used. In this case, these two steps are manually performed.

It is worth highlighting that the filling process can be performed manually, or even integrated in the automatic process. The last option would imply that the conductivity sensor would not be necessary for detecting the liquid. In addition, the mixing temperature and the required degree of transparency could be modified, if necessary. Finally, although the electrophoresis process is disconnected from the agarose fabrication, they can be joined by software. In order to do, automatic pipettes, and the integration of the actuation of the lateral electrodes are necessary. This is possible, but the low-cost nature of the device is lost. However, it could be interesting for large laboratories.

## 5. Results and Discussion

Before performing the experiment to prepare the agarose gel and define a program to control the process, the characterisation of the sensors and actuators is required.

**Figure 5:**
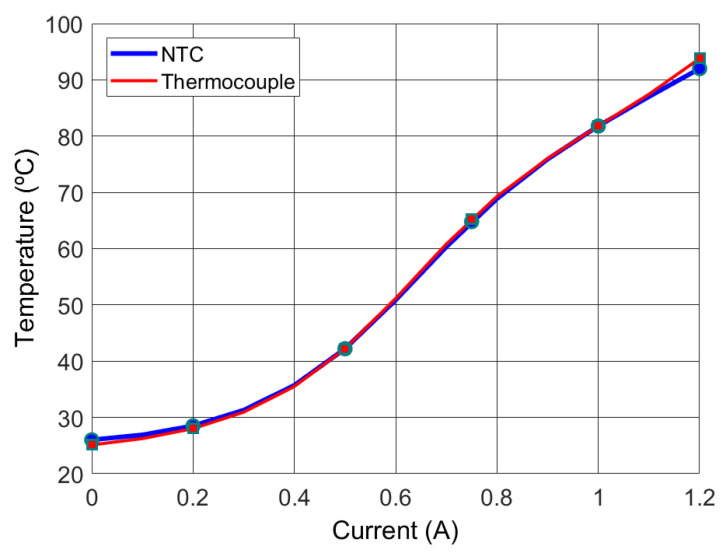
Temperature of both the agarose-TAE solution (thermocouple) and the NTC thermistor as a function of the current.

The microheater characterisation consists of relating the temperature of the agarose-TAE solution with the temperature of the negative temperature coefficient (NTC) thermistor. In order to do so, a thermocouple is used for measuring the temperature of the liquid of the cavity (agarose-TAE solution). In addition, the current supplied to microheater has to be defined. The results for the microheater can be seen in Figure 5.

The temperature for performing the agarose gel is chosen to be a set point of 80 ${}^{\circ }$C. This implies a thermistor resistance of 1680 Ω. The circuit to control the temperature is a voltage divider, so the voltage of the thermistor is applied to an analogue pin of the microcontroller (Figure 4). In addition, a driver (TC4427CPA) and a MOSFET (nMOS IRFB4227PBF) are required to supply a controlled current to the microheater.

The temperatures on the two sides of the PCB substrate are quite different. The surface at low temperature is in contact with the agarose-TAE solution, and the opposite side is in contact with air. Similarly to this effect, the temperature in the centre of the microheater is higher than the temperature where the NTC sensor is placed, both of them being on the bottom side of the PCB. These effects compensate for each other, and the temperatures of the NTC and the liquid (thermocouple) are very close, as can be seen in Figure 5. The authors have considered that the temperatures are the same for this device; however, for a different design of the device, this assumption could be incorrect. Three tests were performed to obtain the behaviour depicted in Figure 5. All of them showed a very similar behaviour, with the electrical current ranging between 0.95 and 1 A for a temperature of 80 ${}^{\circ }$C. After that, the final experiments (automatic working) showed the same behaviour. Regarding the boundary conditions, the thermocouple was placed inside the liquid at approximately half its height. In addition, the room temperature was 25–26 ${}^{\circ }$C. Measures to avoid convection were not used, and the conditions of the room imply natural convection.

**Figure 6:**
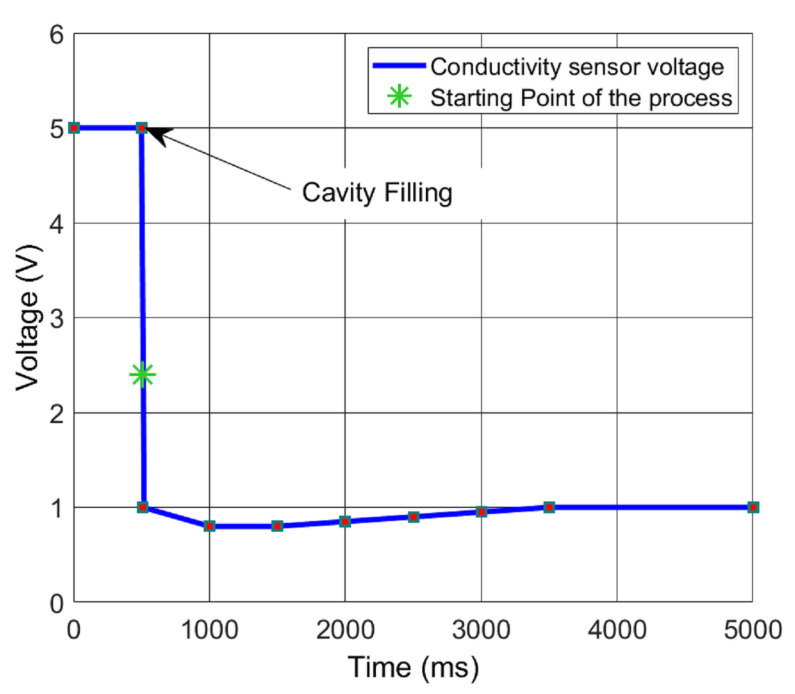
The output of the voltage divider as a function of the time is shown. The starting point is marked using an asterisk.

The characterisation of the interdigitated conductivity sensor is simple to perform. The objective is to obtain a value of the conductivity to define a starting point of the process. In this case, the conductance before filling the cavity with the TAE buffer is 0 S. When the liquid is in the cavity, the conductance rapidly decreases up to 0.04 mS, which corresponds to a resistance of 20 kΩ. This value is chosen to define the starting point of the process, that is, the process starts when the conductivity sensor reaches 0.04 mS. The electronic circuit is a voltage divider. Finally, this characterisation is carried out using an oscilloscope (Tektronix TDS 2012B, single seq. “falling” procedure); Figure 6.

**Figure 7:**
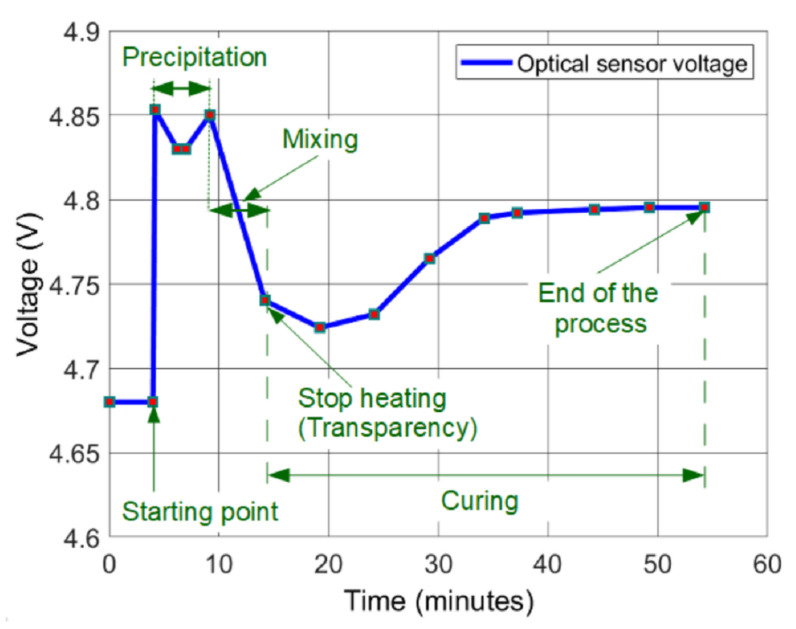
Different values of the optical sensor voltage as a function of the time.

The characterisation of the optical sensor consists of measuring the degree of transparency of the agarose-TAE solution; Figure 7. In order to do so, another voltage divider is used, where the voltage of the LDR is measured. In this case, the agarose gel performed is 2.5% *w*/*v* in the TAE buffer. The choice of the final transparency of the mixture is defined, taking into account the expertise of the authors.

The initial value of the optical sensor is 4.68 V before pouring the TAE buffer. After that, this value increases up to 4.85 V when the powder goes to the surface of the liquid. Then, the powder starts to precipitate and a slight transparency is achieved. When the powder finishes precipitating, that transparency is lost. At this point, the mixing starts, and the liquid begins to become transparent. Therefore, the voltage decreases. The final transparency is reached at 4.74 V, and the microheater is disabled. Later on, the liquid continues increasing the transparency during the cooling down. Then, the agarose starts curing. Once the transparency is lost again, the process finishes at V = 4.795 V. All these values have to be taken into account for programming the microcontroller (ATMega380P).

The process for preparing the agarose gel takes 40 min from the beginning, where the first seconds are used for filling with the TAE buffer, and the last seconds are for removing the PMMA structure. The rest of the process is automatic, where the mixing time is 10 min and the curing time is about 30 min.

**Figure 8:**
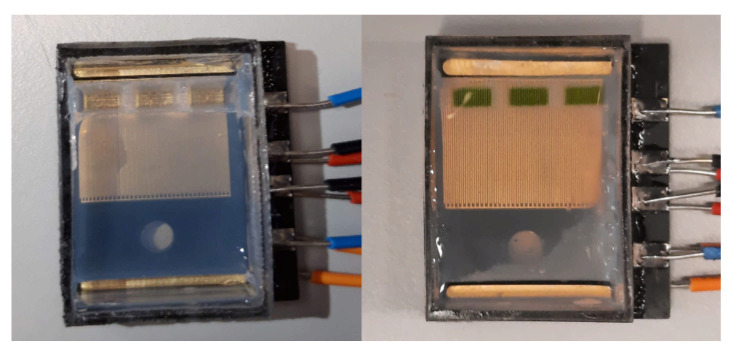
The result of the mixing and curing after removing the structure can be seen. In addition, the wells are filled with liquids.

Once the sensors and actuators are characterised and the microcontroller is programmed, the experiments are carried out. The resulting agarose gel after both the mixing and the curing is shown in Figure 8. In addition, the wells loaded with liquids can be seen.

**Figure 9:**
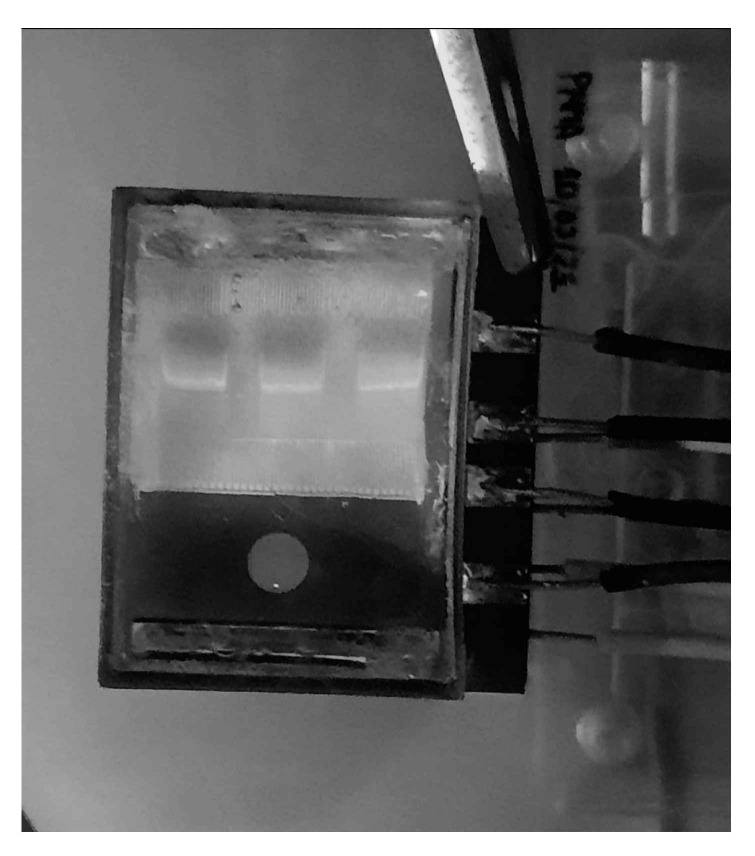
Bands obtained after migrating the DNA by electrophoresis.

The device is checked with DNA in order to verify the correct migration of DNA along the agarose gel; Figure 9. This is important to analyse the homogeneity of the agarose gel. In order to do so, the electrophoresis is performed at 40 V with a required current of 20 mA. The negative and positive electrodes are shown in Figure 1 with black and red arrows, respectively.

As can be seen, three parallel bands are obtained after the DNA migration by electrophoresis. These bands are not appreciably deformed in the agarose gel. Therefore, the homogeneity is acceptable.

The experimental results show good behaviour of the prototype under the chosen parameters. A balance between bubble generation and rapid migration have to be taken into account. In this respect, the generation of bubbles during the electrophoresis for voltages higher than 50 V is very quick. In this case, the diameter of many generated bubbles is very high, about 2 mm. This is due to the fact that the rapid bubble generation in a small cavity implies that the small bubbles coalesce to form bigger ones. On the other hand, the migration at 40 V is rapid enough—in this case 7 min—so that the gold electrodes support the operation. This migration rate can be improved if the concentration of agarose in the fabricated gel decreases, for example 1%. Regarding the location of the transparent film, it must be placed on the top surface of the PCB. Otherwise, it does not support the temperature of the microheater.

It is worthy to highlight that the central band of the Figure 9 is not deformed after the electrophoresis. However, the two other bands are slightly deformed close to the lateral walls. This effect is due to the fact that the final agarose gel is slightly thicker in that area because the surface tension of the liquid is not negligible. Therefore, the resistance to the migration is higher. We are planning to reduce the dimensions of the wells in future developments of the prototype, in order to increase the distance between them and the walls, and to minimise the deformations.

## 6. Conclusions

A semi-automatic prototype of a single-use device for agarose gel preparation and electrophoresis is described. It includes a conductivity sensor for detecting the agarose-TAE solution in order to start the automatic process. In addition, the device has an integrated microheater and a NTC thermistor for controlling the mixing temperature. Moreover, an optical sensor is used for measuring the degree of transparency. The device is lab-on-PCB fabricated, using commercial PCB substrates and thermoplastics. This fact implies low cost, fast analysis, low sample consumption, integration capability and mass production.

It is intended as an integrable functional module of a more complex system for DNA amplification and detection, using qualitative PCR, or even a device itself, to perform more controllable electrophoresis in conventional laboratories, minimizing the human factor.

The proposed lab-on-PCB is intended to be integrated with lab on chip thermocyclers and fluorescence detection systems for developing automatic PCR devices. The whole automatic system will include the DNA amplification, agarose gel fabrication, electrophoresis and detection. The application of this device is point-of-care diagnosis based on qualitative PCR. Finally, the proposed system can be used for conventional PCR procedures as an independent lab-on-PCB device. In addition, the device can be fabricated with smaller dimensions for a higher integration on lab on chip, or even larger ones for conventional multiwells agarose gels.

## Figures and Tables

**Table 1:** Whole process sequence for agarose gel preparation and electrophoresis.

	Agarose preparation	
Step	Function	Automatic
1	Filling of the cavity	First step
2	Mixing	Yes
3	Curing detection	Yes
4	Removing of the auxiliary structure	No
	Electrophoresis	
Step	Function	Automatic
5	DNA loading	No
6	Electrophoresis	No

## Data Availability

Data is contained within the article.

## Abbreviations

The following abbreviations are used in this manuscript:

MDPI	Multidisciplinary Digital Publishing Institute
NTC	Negative Temperature Coefficient
LDR	Light Dependent Resistor
PCR	Polymerase Chain Reaction
PCB	Printed Circuit Board
